# Amorphization–Densification Coupling Governs Hardness Enhancement in SPS-Consolidated Al–Fe–Nb–(Ni,Ti) Metastable Alloys

**DOI:** 10.3390/ma19122628

**Published:** 2026-06-18

**Authors:** Nguyen Thi Hoang Oanh, Nguyen Hoang Viet

**Affiliations:** School of Materials Science and Engineering, Hanoi University of Science and Technology, No 1 Dai Co Viet, Mai, Hanoi 100000, Vietnam; oanh.nguyenthihoang@hust.edu.vn

**Keywords:** Al-rich alloys, mechanical alloying, amorphization, crystallization, spark plasma sintering, microhardness

## Abstract

**Highlights:**

- Nb, Ni, and Ti additions progressively promote amorphization in the mechanically alloyed Al_82_Fe_14−x_Nb_2_(Ni/Ti)_x_ system.
- The quinary Al_82_Fe_12_Nb_2_Ni_2_Ti_2_ alloy shows the strongest amorphization tendency, driven by higher ΔS_mix_ and more negative ΔH_mix_.
- SPS at 500 °C yields amorphous–nanocrystalline composites containing Al_13_Fe_4_ and Al_3_Nb nanophases.
- Maximum hardness (445.4 HV) is achieved in Al_82_Fe_14_Nb_2_Ni_2_, not in the most amorphous alloy.
- Hardness response requires kinetic balance between amorphous retention, nanocrystallization, and densification efficiency.

**Abstract:**

The coupled effects of Ni and Ti additions on amorphization, spark plasma sintering (SPS) response, and hardness evolution were investigated in Al-rich Al–Fe–Nb-based metastable alloys. Mechanically alloyed Al_82_Fe_14_Nb_2_Ni_2_, Al_82_Fe_14_Nb_2_Ti_2_, and Al_82_Fe_12_Nb_2_Ni_2_Ti_2_ powders showed progressive loss of long-range order, with the quinary alloy exhibiting the strongest amorphization tendency, consistent with its higher configurational entropy (5.420 J·mol^−1^·K^−1^) and more negative mixing enthalpy (−9.36 kJ·mol^−1^). SPS displacement analysis revealed that primary displacement contribution occurs during heating and is progressively limited by crystallization-induced stiffening. Consolidation at 500 °C produced amorphous–nanocrystalline composites containing Al_13_Fe_4_ and Al_3_Nb, whereas increasing the temperature to 550 °C promoted further devitrification. The highest hardness, 445.4 HV, was obtained for Al_82_Fe_14_Nb_2_Ni_2_, despite its lower amorphous-forming ability than the quinary alloy. This demonstrates that hardness is controlled not by maximum amorphization, but by the kinetic balance between amorphous retention, fine intermetallic precipitation, and densification efficiency. The results identify SPS as a coupled densification–transformation route for designing high-strength Al-based amorphous–nanocrystalline alloys.

## 1. Introduction

Metallic glasses are metastable alloys in which the absence of long-range crystalline order suppresses conventional dislocation-mediated plasticity and creates structure–property relationships that differ fundamentally from those of crystalline metals [1,2,3,4]. Within this class, Al-based amorphous, nanoquasicrystalline, and amorphous–nanocrystalline alloys are especially attractive because they combine the intrinsic low density of Al with high specific strength, high hardness, wear resistance, and corrosion resistance [5,6,7,8]. Their metastable character also opens a microstructural design space that is not available in conventional precipitation-hardened Al alloys. In particular, partial devitrification of an amorphous precursor can generate ultrafine crystalline phases or nanoscale crystalline/amorphous composites, providing strengthening routes based on amorphous retention, nanocrystal dispersion, and interfacial constraints rather than solely on equilibrium precipitate hardening [5,9]. However, preparing fully amorphous Al-rich alloys remains difficult because many Al-based systems are marginal glass formers with limited glass-forming ability (GFA) [10,11,12]. This limitation is commonly associated with rapid crystallization kinetics, high atomic mobility in Al-rich melts or mechanically activated powders, and a narrow supercooled-liquid or processing window before crystallization [13,14]. Mechanical alloying (MA) is therefore a particularly useful route for Al-rich metastable alloys because it bypasses the severe cooling-rate requirements of melt processing and produces non-equilibrium structures through repeated cold welding, fracturing, rewelding, defect accumulation, and atomic-scale mixing [15,16,17,18,19].

The amorphization of multicomponent Al-rich systems is governed by coupled thermodynamic, kinetic, and topological factors. Negative mixing enthalpy promotes heteroatomic bonding and chemical short-range ordering, while atomic-size mismatch frustrates efficient crystalline packing and increases local lattice distortion [13,14,20]. Configurational complexity further reduces the relative stability of simple ordered phases by increasing compositional disorder, especially in multicomponent alloys where several competing local environments coexist [20,21]. Recent descriptor-based and data-driven studies have shown that mixing enthalpy, atomic-size mismatch, configurational entropy, and related composition descriptors can be correlated with phase selection in amorphous and complex concentrated alloys [20,21,22]. This framework is particularly relevant for Al-rich systems, where small compositional changes can shift the pathway among nanocrystalline refinement, amorphization, and intermetallic precipitation.

Among Al-rich metallic-glass-forming systems, Al-Fe-based alloys are attractive because Fe is inexpensive, abundant, and capable of contributing to strengthening, magnetic response, and thermal stability [23]. Previous studies have shown that the structural evolution of mechanically alloyed Al-Fe powders is highly sensitive to minor transition-element additions that modify atomic-size mismatch, chemical affinity, and local ordering tendency [24,25]. In Al_82_Fe_16_TM_2_ (TM = Ti, Ni, Cu) alloys, the choice of TM element strongly affects the extent of structural disordering during milling and the resulting magnetic behavior [24]. Similarly, studies on Al-Fe-Y alloys have demonstrated that Ni and Ti additions influence dissolution behavior, transient solid-solution formation, GFA, and magnetic response [25]. These findings indicate that minor alloying additions actively regulate the kinetic and thermodynamic pathways of metastable structure formation.

Ni is generally associated with strong negative mixing enthalpy with Al and Fe, which accelerates interdiffusion at particle interfaces and promotes the formation of chemically disordered solid solutions during milling. Ti, by contrast, has a larger atomic radius and introduces greater local lattice distortion, topologically frustrating crystalline packing and impeding long-range ordering independently of chemical affinity. These distinct mechanisms—chemical-affinity-driven mixing for Ni and topological frustration for Ti—imply that their combined addition in the quinary alloy may act synergistically to enhance amorphization beyond what either element achieves alone. The formation of fully amorphous Al_82_Fe_14_Ni_4_ powders by MA confirms that Ni-bearing Al-Fe alloys can reach a highly disordered state under sufficiently intense milling conditions [16]. More recently, Nb-containing Al-Fe powders have been shown to undergo pronounced nanocrystalline-to-amorphous evolution during high-energy ball milling, highlighting the role of Nb in destabilizing crystalline phases and improving structural tunability in Al-rich metastable alloys [26,27].

However, direct comparative studies of closely related Al-rich Al-Fe-Nb compositions containing Ni, Ti, or simultaneous Ni-Ti additions remain limited [25]. Such a compositional series is useful because it allows the effects of chemical affinity, atomic-size mismatch, and multicomponent interactions to be more clearly separated. Ni and Ti can both promote alloying and structural disorder, but they differ in atomic radius, bonding preference, and interaction with Al- and Fe-rich local environments [25]. When combined with Nb, these differences are expected to influence not only amorphization during milling but also phase selection during spark plasma sintering (SPS). Related Al-Fe-Ti-Ni studies have shown that metastable amorphous phases may transform into nanoscale intermetallic compounds such as Al_13_(Fe,Ni)_4_ and Al_3_Ti, indicating that short-range chemical order established during non-equilibrium processing controls subsequent microstructural evolution [28]. Here, the metastable states of interest are not equilibrium Al-based phases, but MA-induced amorphous-dominant and nanocrystalline/amorphous structures, together with SPS-derived partially devitrified amorphous–nanocrystalline composites containing retained amorphous regions and nanoscale Al_13_Fe_4_-, Al_3_Nb-, and Al_3_Ti-type intermetallic phases.

Beyond powder synthesis, the consolidation of amorphous or amorphous-dominant powders into dense bulk forms is a critical step because densification and crystallization occur as competing but strongly coupled processes during SPS [29,30,31,32,33]. Rapid heating, electric-current assistance, and applied pressure can promote particle rearrangement, viscous or plastic flow, neck formation, pore closure, and interparticle bonding [31,34,35]. At the same time, thermal activation may induce structural relaxation and devitrification, leading to the nucleation and growth of crystalline or intermetallic phases [29,33,36]. Once crystallization becomes extensive, the compact stiffens, atomic mobility decreases, and further densification may be restricted, resulting in residual porosity or heterogeneous bonding [33,36]. Therefore, amorphous retention, densification efficiency, and mechanical properties are governed by the coupled effects of sintering temperature, pressure, heating rate, and holding time [31,36,37,38,39,40,41,42]. Insufficient thermal exposure may lead to poor interparticle bonding, whereas excessive temperature or prolonged holding can promote crystallization and coarsening, reducing the strengthening contribution of the retained amorphous matrix [33,36,42]. Accordingly, SPS should be regarded not simply as a densification route, but as a transformation-assisted microstructural tuning process in which the metastable state produced by MA is partially retained and partially converted into a mechanically effective amorphous–nanocrystalline composite [31,32,33,36,43,44,45].

This study aims to clarify how Ni and Ti additions affect the amorphization behavior, SPS consolidation response, microstructure, and hardness of mechanically alloyed Al-Fe-Nb-based alloys. Particular emphasis is placed on connecting thermodynamic descriptors with XRD phase evolution, SPS-induced partial crystallization, densification behavior, and the resulting hardness response. The central hypothesis is that maximum amorphization alone does not guarantee maximum hardness; instead, the highest hardness emerges from a kinetic balance between amorphous retention, controlled fine intermetallic formation, and efficient densification during SPS.

## 2. Materials and Methods

Elemental powders of aluminum (Al), iron (Fe), niobium (Nb), titanium (Ti), and nickel (Ni) with a purity of ≥97% were mixed into three different compositions of Al_82_Fe_14_Nb_2_Ni_2_, Al_82_Fe_14_Nb_2_Ti_2_, and Al_82_Fe_12_Nb_2_Ni_2_Ti_2_, as shown in Table 1. Milling was performed using a planetary ball mill (AGO-2 type) in hardened steel vials and tungsten carbide (WC) balls at 350 rpm, with a ball-to-powder weight ratio of 20:1. To prevent excessive cold welding, 50 mL n-hexane was used as a process control agent. All experiments were conducted under an argon atmosphere. To limit localized temperature increases during milling, the milling container was actively cooled by circulating water flowing through its jacket throughout the milling process. Powder samples were collected after 1–20 h of milling.

The as-milled powders were consolidated by spark plasma sintering using a LABOX-625F (Sinterland Corporation, Tokyo, Japan). Sintering was carried out at 500 °C under 500 MPa for 5 min for all alloys, and the Al_82_Fe_12_Nb_2_Ni_2_Ti_2_ alloy was additionally sintered at 550 °C. The 500–550 °C window was selected to promote densification while limiting excessive crystallization. This choice is consistent with previous SPS studies on Al-based amorphous or glass-derived powders, which indicate that effective consolidation is typically achieved near the onset of crystallization: temperatures that are too low may limit interparticle bonding and densification, whereas excessive temperatures can accelerate devitrification, intermetallic coarsening, and loss of the amorphous contribution [29,31,33]. Accordingly, 500 °C served as the baseline condition, while 550 °C was used to evaluate whether moderate additional crystallization in the most amorphous alloy could improve hardness. No SPS experiments were performed below 500 °C or above 550 °C; therefore, the present conditions define a practical processing window rather than a fully explored sintering range. No thermal analysis (DSC or DTA) was performed to determine the crystallization onset temperature of the present alloys; the selected SPS window should therefore be regarded as an empirically motivated processing range consistent with related Al-based systems rather than a thermodynamically justified optimum.

Vickers microhardness measurements were performed using a Vickers diamond indenter under an applied load of 50 gf and a dwell time of 10 s. For each sample, five indentations were carried out, including one indentation at the center of the sample and four indentations symmetrically distributed around the region of interest, with sufficient spacing between adjacent indentations to avoid overlap of the plastic deformation zones. Indentations affected by surface defects, edge effects, cracking, or irregular indentation geometry were excluded from the analysis. The reported hardness value was calculated as the arithmetic mean of the valid measurements, and the standard deviation was determined from the same dataset using Excel and independently checked by Python (v 3.12.13).

Phase analysis was performed by X-ray diffraction with an X’Pert PRO Powder Diffractometer (Malvern Panalytical, Malvern, UK). Microstructures were observed using a HITACHI TM4000 PLUS instrument (Hitachi High-Tech Corporation, Tokyo, Japan). Vickers hardness was measured on polished surfaces, and the reported values were averaged from multiple indentations.

The amorphization tendency of the investigated alloys was evaluated by calculating key thermodynamic and topological parameters: configurational mixing entropy (ΔS_mix_), enthalpy of mixing (ΔH_mix_), and atomic-size mismatch (δ). Based on the Miedema macroscopic model, these parameters were computed via a custom Python script using data sourced from Takeuchi and Inoue [46]. Detailed calculation procedures and the full source code are available in Table S1 and Code S1 of the Supplementary Materials, respectively.

## 3. Results and Discussion

### 3.1. Phase Evolution During Mechanical Alloying

**Figure 1:**
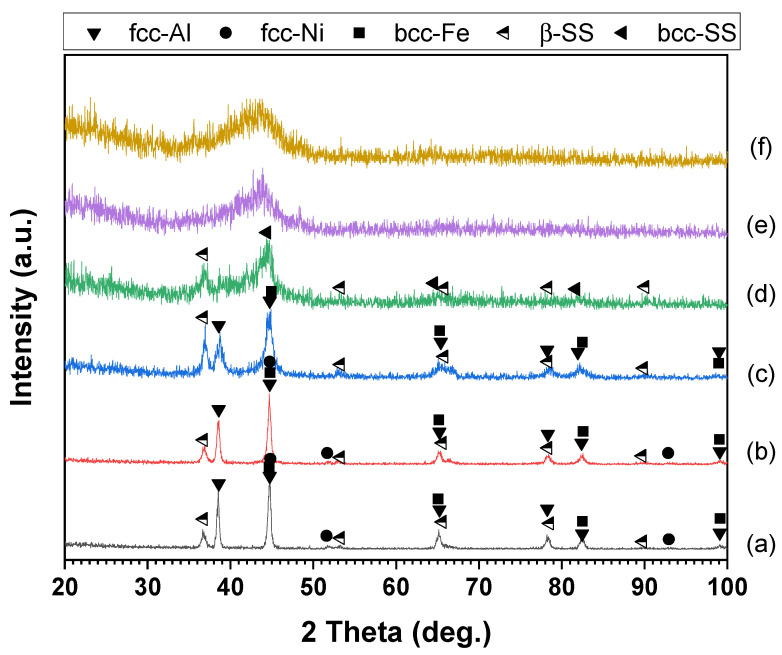
X-ray diffraction patterns of mechanically alloyed Al_82_Fe_14_Nb_2_Ni_2_ powders milled for different durations: (**a**) 1 h, (**b**) 2 h, (**c**) 5 h, (**d**) 10 h, (**e**) 15 h, and (**f**) 20 h.

**Figure 2:**
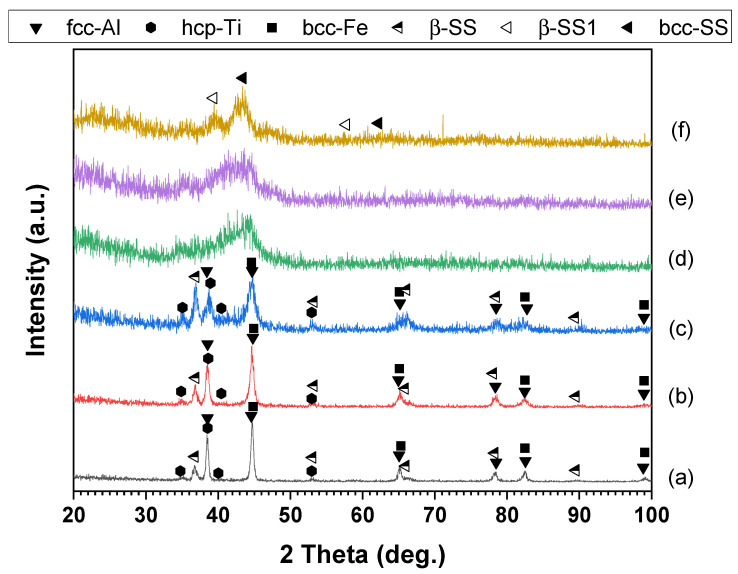
X-ray diffraction patterns of mechanically alloyed Al_82_Fe_14_Nb_2_Ti_2_ powders milled for different durations: (**a**) 1 h, (**b**) 2 h, (**c**) 5 h, (**d**) 10 h, (**e**) 15 h, and (**f**) 20 h.

**Figure 3:**
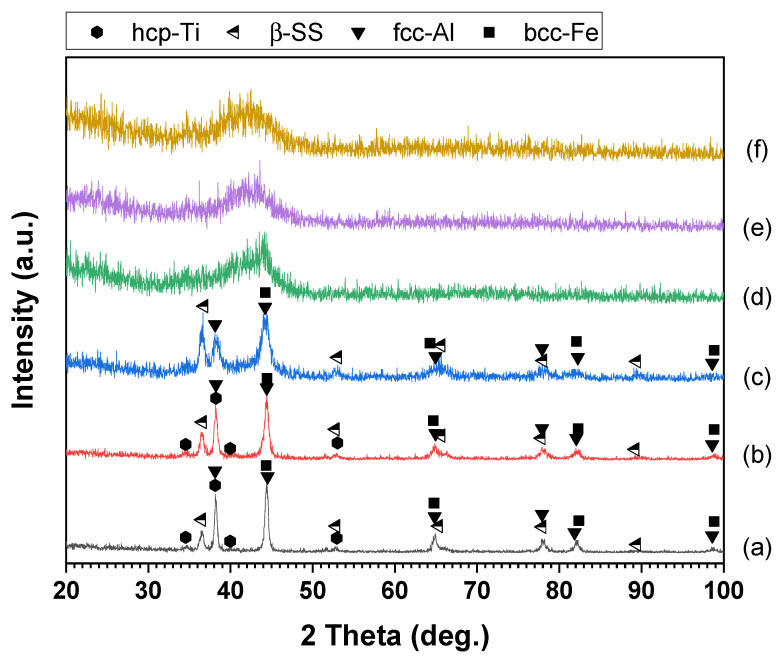
X-ray diffraction patterns of mechanically alloyed Al_82_Fe_12_Nb_2_Ni_2_Ti_2_ powders milled for different durations: (**a**) 1 h, (**b**) 2 h, (**c**) 5 h, (**d**) 10 h, (**e**) 15 h, and (**f**) 20 h.

The XRD patterns of Al_82_Fe_14_Nb_2_Ni_2_, Al_82_Fe_14_Nb_2_Ti_2_, and Al_82_Fe_12_Nb_2_Ni_2_Ti_2_ powders milled for 1–20 h are shown in Figure 1, Figure 2 and Figure 3. For all compositions, increasing milling time leads to peak broadening, intensity reduction, and the gradual formation of a broad diffuse halo at approximately 2θ = 38–45°. These features indicate crystallite refinement, severe lattice strain, and progressive loss of long-range atomic order, which are characteristic signatures of MA-induced amorphization in Al-Fe-based systems [16]. Peak assignments and crystallographic data for all identified phases, including elemental and intermetallic constituents, are summarized in Table S2 of the Supplementary Materials.

For Al_82_Fe_14_Nb_2_Ni_2_ (Figure 1), the powders milled for 1–5 h still show distinct reflections associated with fcc-Al, bcc-Fe, fcc-Ni and the β-solid solution (β-SS, attributed to an Nb-rich multicomponent solid solution), indicating that the structure remains largely crystalline at the early milling stage. By 10 h, fcc-Al, bcc-Fe, and fcc-Ni reflections are markedly weakened, and only residual peaks corresponding to a β-SS and a bcc-type solid solution (bcc-SS) persist alongside a developing broad diffuse background. Beyond 15 h, the pattern becomes dominated by diffuse maxima. This evolution confirms the formation of an amorphous-dominant structure. Compared with Al_82_Fe_14_Ni_4_, for which complete disappearance of crystalline peaks requires approximately 60 h of milling [16], the faster structural disordering observed here suggests that Nb promotes crystalline destabilization and accelerates amorphization.

Al_82_Fe_14_Nb_2_Ti_2_ shows a similar but slightly faster transformation (Figure 2). At 5 h, the diffraction peaks are already broader and weaker than those of the Ni-containing alloy, and by 10 h, the pattern becomes largely halo-like. This behavior indicates that Ti is more effective than Ni in promoting amorphization under the present milling conditions. The observation agrees with previous results for Al_82_Fe_16_TM_2_ alloys, where minor TM additions were shown to strongly affect structural disordering, thermal stability, and crystallization behavior.

Among the three alloys, Al_82_Fe_12_Nb_2_Ni_2_Ti_2_ exhibits the most pronounced amorphization tendency based on XRD peak evolution (Figure 3). Even at short milling times, the peaks are weaker and broader, and after 10–20 h, the patterns are nearly halo-dominated, with only minor crystalline remnants. The enhanced amorphization response is attributed to the synergistic effect of Nb, Ni, and Ti additions, which increases chemical complexity and configurational disorder while suppressing the nucleation and growth of ordered phases. Compared with Al_82_Fe_16_Ce_2_ and Al_82_Fe_14_Mn_2_Ce_2_, which require approximately 40 h of milling to reach full amorphization [23], the present quinary alloy reaches a halo-dominant state within 10–20 h. This confirms that the simultaneous addition of Nb, Ni, and Ti is highly effective in accelerating amorphization during MA.

### 3.2. Thermodynamic Factors Governing Amorphization

To interpret the amorphization behavior of the three alloys, key thermodynamic and topological parameters were evaluated and correlated with the XRD results. The configurational mixing entropy (ΔS_mix_), which reflects the degree of chemical disorder, was calculated using [22,47,48,49]:

(1) $${∆S}_{mix}=-R\sum_{i=1}^{n}c_{i}lnc_{i}$$

where R is the gas constant and xᵢ is the atomic fraction of the i-th element. A higher ΔS_mix_ favors disordered solid solutions and increases the tendency toward amorphization by enhancing configurational randomness [22,47,48,49]. In multicomponent systems, configurational entropy contributes to lowering the Gibbs free energy, ΔG_mix_ = ΔH_mix_ − T × ΔS_mix_, thereby supporting the stabilization of disordered phases [20]. Although Al-rich alloys generally show lower entropy than equiatomic high-entropy alloys, values approaching 0.5–0.8 R can still meaningfully contribute to disorder stabilization when combined with favorable enthalpy and atomic-size effects:

ΔG_mix_ = ΔH_mix_ − T × ΔS_mix_ (2)

(3) $${∆H}_{mix}=\sum_{i=1,\;i\neq j}^{n}4H_{ij}c_{i}c_{j}$$

where ΔHᵢⱼ is the binary mixing enthalpy between elements A and B. Negative ΔH_mix_ values indicate favorable heteroatomic interactions, which promote atomic-scale mixing and suppress the nucleation of ordered crystalline phases [50]. Data-driven and CALPHAD-based studies have further identified ΔH_mix_ as one of the important descriptors governing phase formation in multicomponent alloys. Moderately negative values, particularly in the range of approximately −10 to −30 kJ/mol, are often associated with enhanced GFA because strong chemical affinity and short-range ordering frustrate long-range crystallization [27,50]:

(4) $$\delta =\sqrt{\sum_{i=1}^{n}c_{i}{\left(r_{i}-\bar{r}\right)}^{2}}\times 100\%$$

where rᵢ is the atomic radius of element i and $\bar{r}$ is the average atomic radius. A larger δ implies greater lattice distortion, which hinders atomic diffusion and long-range ordering and therefore favors amorphization. Reported descriptor-based phase-selection criteria indicate that solid-solution formation is commonly favored when δ is below about 6.6%, whereas larger δ combined with sufficiently negative ΔH_mix_ may promote amorphous phase formation or intermetallic competition [20,51]. In practical mechanically alloyed Al-based systems, δ values close to 5% can still accelerate disordering because severe plastic deformation continuously introduces defects and stored energy. Together, ΔS_mix_, ΔH_mix_, and δ provide a consistent framework for understanding amorphous phase formation in the investigated alloys. ΔS_mix_ reflects configurational disorder, ΔH_mix_ represents the chemical driving force for heteroatomic mixing, and δ quantifies lattice distortion. Their combined effects determine whether mechanical alloying promotes crystalline refinement, supersaturated solid-solution formation, or amorphous-dominant structures.

The calculated parameters in Table 2 are consistent with the XRD-observed phase evolution. All alloys exhibit negative ΔH_mix_ values (−7.92 to −9.36 kJ/mol), confirming favorable chemical interactions and a clear driving force for alloying. These values fall within the typical range reported for Al-TM and multicomponent alloy systems, where a more negative ΔH_mix_ is generally associated with stronger heteroatomic bonding and enhanced amorphization tendency [20,27]. In kinetic terms, negative ΔH_mix_ also facilitates interdiffusion during MA by promoting local chemical mixing at newly created interfaces and reducing the likelihood of re-establishing stable crystalline regions [49]. The configurational entropy values are 4.94 J/mol·K for the two quaternary alloys and 5.420 J/mol·K for the quinary alloy, corresponding to approximately 0.60–0.70 R. These values are typical for Al-rich multicomponent alloys [27,48]. Although they are lower than those of equiatomic high-entropy alloys, the increase in ΔS_mix_ contributes to disorder stabilization when combined with negative ΔH_mix_ and sufficient lattice distortion [27,50]. Accordingly, the higher ΔS_mix_ of Al_82_Fe_12_Nb_2_Ni_2_Ti_2_ supports its stronger amorphization tendency. The atomic-size mismatch parameter δ ranges from 4.73 to 4.98%, placing the alloys in the transition region between solid-solution and amorphous regimes [50,51]. This indicates that atomic-size mismatch alone is insufficient to guarantee amorphization, but it becomes effective under the severe deformation conditions of MA. Repeated ball impacts generate high stored energy, lattice strain, and defect density, progressively destabilizing the crystalline state. Therefore, amorphization in the present alloys should be understood as a coupled thermodynamic–kinetic process: ΔH_mix_ promotes chemical mixing, ΔS_mix_ stabilizes disorder, and δ, together with defect accumulation, accelerates the collapse of long-range order [27,50,51].

The combined effects of ΔH_mix_, ΔS_mix_, and δ can be rationalized within a unified processing–structure–property framework. These three descriptors collectively govern the degree of amorphization achieved during mechanical alloying: ΔH_mix_ promotes chemical mixing and short-range ordering, ΔS_mix_ stabilizes configurational disorder, and δ introduces topological frustration that suppresses long-range crystallization. As a result, their convergence defines the amorphous-forming ability of the alloy system. However, the present results demonstrate that maximum amorphization does not directly correspond to optimal mechanical performance. Instead, the amorphous state serves as a precursor that determines the subsequent response during spark plasma sintering (SPS). During SPS, the metastable amorphous structure evolves through a coupled densification-devitrification process. A highly amorphous powder promotes enhanced densification due to increased plasticity and defect-assisted diffusion, but excessive amorphous stability may delay beneficial nanocrystallization. Conversely, insufficient amorphization leads to premature crystallization, restricting densification and resulting in microstructural heterogeneity. Therefore, maximum hardness in the studied parameter set emerges from a kinetic balance between amorphous retention, partial devitrification into fine intermetallic precipitates, and densification efficiency. This establishes a convergence pathway: (ΔH_mix_ − ΔS_mix_ − δ) → amorphization → SPS densification/devitrification → maximum hardness, which provides a descriptor-guided strategy for designing high-performance Al-based amorphous–nanocrystalline alloys.

### 3.3. Phase Evolution During SPS Consolidation

**Figure 4:**
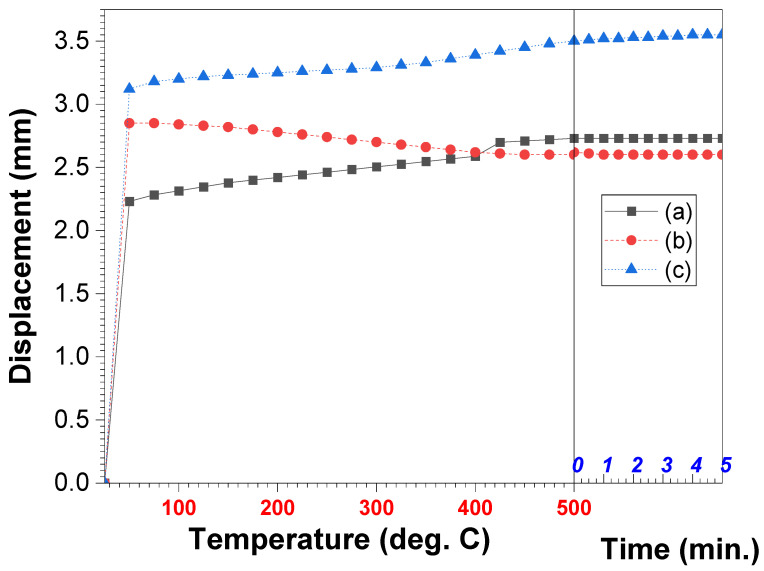
SPS displacement curves: (**a**) Al_82_Fe_14_Nb_2_Ni_2_, (**b**) Al_82_Fe_14_Nb_2_Ti_2_, and (**c**) Al_82_Fe_12_Nb_2_Ni_2_Ti_2_ powders sintered at 500 °C.

The displacement curves in Figure 4 reflect the densification behavior of the mechanically alloyed powders during SPS. All samples exhibit a sharp initial increase, mainly due to particle rearrangement, plastic deformation at contacts, and collapse of weak agglomerates under pressure. Subsequently, displacement increases more gradually up to 500 °C, indicating densification governed by thermally assisted diffusion, viscous or plastic flow of defect-rich particles, and progressive bonding at particle contacts. This behavior is consistent with electric-current-assisted sintering, where pressure, pulsed current, and rapid heating enhance interparticle contact while limiting grain growth [31,34,35]. A clear composition dependence is observed. The Al_82_Fe_14_Nb_2_Ni_2_ compact shows a steady increase in displacement, indicating stable densification. The Al_82_Fe_14_Nb_2_Ti_2_ alloy exhibits high initial displacement followed by stabilization, suggesting dominant early rearrangement but less efficient subsequent densification, likely due to non-uniform bonding or early crystallization-induced stiffening. In contrast, Al_82_Fe_12_Nb_2_Ni_2_Ti_2_ shows the largest total displacement, reflecting higher compressibility and sinterability associated with its more amorphous character. During the 5 min holding stage at 500 °C, all curves approach a plateau, indicating that the major displacement contribution occurs during heating, with the dwell stage mainly stabilizing the compact. The absence of abrupt displacement changes suggests that any crystallization proceeds gradually, favoring retention of an amorphous–nanocrystalline structure [29,33].

**Figure 5:**
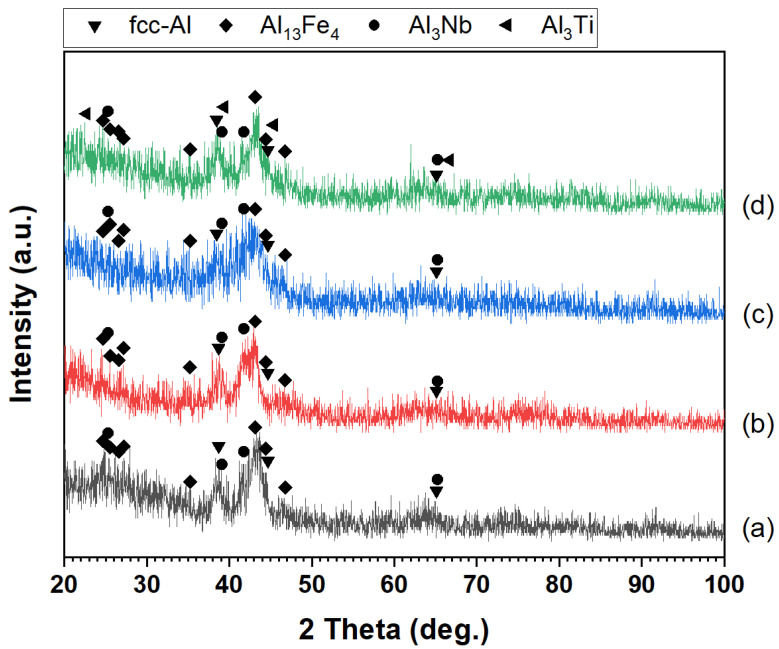
XRD patterns of SPS-consolidated alloys: (**a**) Al_82_Fe_14_Nb_2_Ni_2_, (**b**) Al_82_Fe_14_Nb_2_Ti_2_, and (**c**) Al_82_Fe_12_Nb_2_Ni_2_Ti_2_ powders sintered at 500 °C; (**d**) Al_82_Fe_12_Nb_2_Ni_2_Ti_2_ powders sintered at 550 °C.

The XRD patterns of the three alloys after SPS at 500 °C are shown in Figure 5a–c. All consolidated samples retain a broad diffuse background, indicating that complete crystallization does not occur during the short SPS cycle. However, weak reflections corresponding mainly to Al_13_Fe_4_, Al_3_Nb, and residual fcc-Al are observed, confirming partial devitrification during consolidation. Among the three alloys, Al_82_Fe_12_Nb_2_Ni_2_Ti_2_ shows the broadest and most diffuse post-SPS profile, indicating the lowest apparent crystalline peak intensity after SPS. This behavior is consistent with its higher ΔS_mix_ and more negative ΔH_mix_, both of which are expected to retard long-range atomic rearrangement. In contrast, Al_82_Fe_14_Nb_2_Ni_2_ shows stronger crystalline reflections, indicating more pronounced devitrification. Figure 5d shows that increasing the sintering temperature of Al_82_Fe_12_Nb_2_Ni_2_Ti_2_ to 550 °C increases the intensity of reflections associated with Al_3_Nb, Al_3_Ti, and Al_13_Fe_4_, confirming that higher SPS temperature promotes additional crystallization [31,33]. From a thermodynamic viewpoint, the strong negative interactions of Al with Nb and Ti favor the formation of Al_3_Nb and Al_3_Ti, while pressure and pulsed current during SPS enhance local atomic mobility at particle contacts and promote densification without allowing extensive grain growth [31,34]. Therefore, the 500–550 °C range can be regarded as a compromise window in which densification proceeds while crystallization remains sufficiently constrained. In this interpretation, 500 °C is close to a lower practical consolidation boundary for retaining the amorphous contribution, whereas 550 °C provides a controlled increase in thermal driving force to test whether limited nanocrystallization can improve hardness. The current data show that raising the temperature intensifies crystallization; however, because temperatures below 500 °C and above 550 °C were not examined, the true optimum SPS temperature should be determined in future work through a broader temperature sweep.

Combining Figure 4 and Figure 5 indicates that the SPS response is controlled by the competition between densification and partial devitrification. The larger displacement of the quinary Al_82_Fe_12_Nb_2_Ni_2_Ti_2_ alloy in Figure 4 agrees with its broad post-SPS XRD background in Figure 5, confirming that the amorphous-dominant structure promotes densification while delaying long-range crystallization. Conversely, the stronger crystalline reflections of Al_82_Fe_14_Nb_2_Ni_2_ after SPS indicate that part of the stored energy introduced by mechanical alloying is consumed by controlled intermetallic precipitation during consolidation. Therefore, the displacement curve should not be interpreted only as a measure of final density; it also reflects the temperature-dependent structural state of the powder compact and its ability to densify before crystallization restricts particle rearrangement [29,31].

### 3.4. Microstructural Features of SPS Compacts

**Figure 6:**
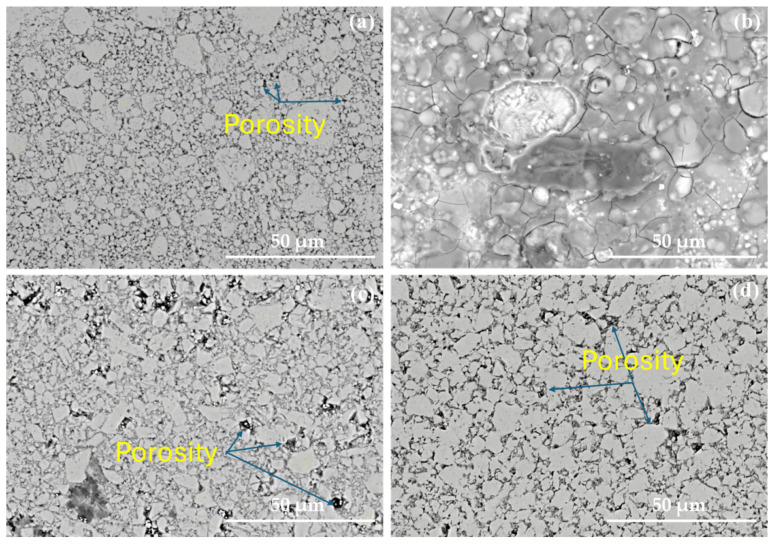
SEM micrographs of SPS-consolidated samples: (**a**) Al_82_Fe_14_Nb_2_Ni_2_ sintered at 500 °C, (**b**) Al_82_Fe_14_Nb_2_Ti_2_ sintered at 500 °C, (**c**) Al_82_Fe_12_Nb_2_Ni_2_Ti_2_ sintered at 500 °C, and (**d**) Al_82_Fe_12_Nb_2_Ni_2_Ti_2_ sintered at 550 °C. Arrows indicate residual porosity.

**Figure 7:**
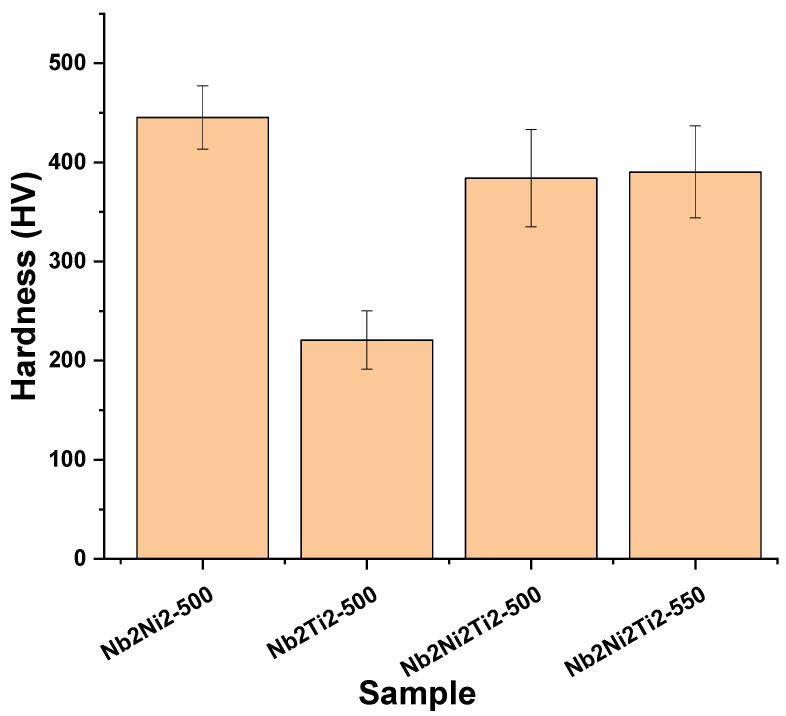
Bar chart comparing the average Vickers hardness (HV) and standard deviation of SPS-consolidated Al_82_Fe_14_Nb_2_Ni_2_, Al_82_Fe_14_Nb_2_Ti_2_, and Al_82_Fe_12_Nb_2_Ni_2_Ti_2_ alloys sintered at 500 °C and 550 °C under 500 MPa for 5 min.

The SEM micrographs of the consolidated samples are shown in Figure 6. The Al_82_Fe_14_Nb_2_Ni_2_ compact sintered at 500 °C (Figure 6a) exhibits the most favorable load-bearing microstructure among the investigated samples, with a relatively continuous bonded matrix and no obvious large pores at the observed scale. Although fine dark interparticle regions are still present, the compact appears sufficiently dense to support the highest hardness measured in Table 3 and Figure 7 (445.4 HV). This indicates that the hardness maximum is associated not only with phase constitution, but also with effective particle bonding and a reduced fraction of microstructural defects. In contrast, the Al_82_Fe_14_Nb_2_Ti_2_ compact (Figure 6b) shows a coarser and more heterogeneous morphology, including locally weakly bonded or cracked regions and non-uniform contrast. This microstructural discontinuity is consistent with the lowest hardness in Figure 7, suggesting that residual defects and heterogeneous consolidation reduce the effective load-bearing area and dominate over any potential strengthening from milling-induced amorphization. The quinary Al_82_Fe_12_Nb_2_Ni_2_Ti_2_ compact sintered at 500 °C (Figure 6c) retains a finer amorphous/nanocrystalline matrix, but visible residual porosity remains, as marked in the image. Its intermediate hardness (384.0 HV) therefore reflects a competition between amorphous/nanocrystalline strengthening and porosity-induced softening. It also aligns with the larger, more stable displacement response in Figure 4, suggesting that the quinary powder is suggested to have maintained a longer densification window before extensive crystallization constrained particle rearrangement and interparticle bonding [29,31]. When the quinary alloy is sintered at 550 °C (Figure 6d), the microstructure remains broadly similar but shows residual porosity together with enhanced crystallization, in agreement with the XRD results in Figure 5d. The slight increase in hardness from 384.0 to 390.3 HV indicates that moderate additional devitrification contributes some hardening, but the remaining pores still limit the attainable hardness. Overall, the combined comparison of Figure 4 and Figure 6 supports the view that higher and more continuous SPS shrinkage generally correlates with improved compact uniformity, whereas early crystallization or heterogeneous particle bonding can leave residual pores or coarse contrast features in the final microstructure [24,32,33]. If devitrification occurs too early or too rapidly, the resulting crystalline framework can restrict viscous flow and trap residual porosity; conversely, delayed crystallization is expected to support more uniform densification [31,33]. It should be noted that the present microstructural analysis is qualitative in nature, as no relative density measurements (e.g., Archimedes’ method) or quantitative porosity analysis (e.g., image analysis) were performed. Therefore, statements regarding densification efficiency and the role of porosity in limiting hardness are based on SEM observations and should be interpreted accordingly. Future work will incorporate systematic density and porosity quantification to establish a more rigorous structure–property relationship.

### 3.5. Hardness and Structure–Property Correlation

The peak hardness of the Ni-containing quaternary alloy significantly exceeds that of conventional Al alloys and approaches that of advanced Al-based glass-derived and nanocrystalline materials [52,53,54]. The average Vickers hardness and corresponding standard deviation of the SPS-consolidated alloys sintered at 500 °C and 550 °C are summarized in Table 3 and visually compared in Figure 7. A unified analysis of Figure 4, Figure 5, Figure 6 and Figure 7 supports a clear processing–structure–property relationship governed by the interplay between densification, porosity, and SPS-induced phase transformation. Al_82_Fe_14_Nb_2_Ni_2_ achieves the highest hardness because it combines a relatively continuous compact microstructure (Figure 6a) with controlled crystallization during SPS. The formation of fine Al_13_Fe_4_- and Al_3_Nb-containing dispersions within an amorphous/nanocrystalline matrix enables effective dispersion strengthening and grain refinement while avoiding excessive coarsening. By contrast, the low hardness of Al_82_Fe_14_Nb_2_Ti_2_ is consistent with its coarser and more heterogeneous microstructure in Figure 6b, which indicates less uniform consolidation and a reduced effective load-bearing area. The quinary alloy exhibits larger shrinkage and stronger amorphous retention, but visible residual porosity in Figure 6c limits its hardness to an intermediate level. Increasing the SPS temperature of the quinary alloy to 550 °C produces only a slight hardness increase, consistent with additional devitrification in Figure 5d, because the remaining porosity continues to constrain the mechanical response. Overall, the hardness hierarchy is best understood as a kinetic and microstructural balance rather than a direct function of amorphous fraction: Al_82_Fe_14_Nb_2_Ni_2_ benefits from balanced densification and partial nanocrystallization, the quinary alloy favors amorphous retention but is limited by residual porosity and insufficient strengthening precipitates, and Al_82_Fe_14_Nb_2_Ti_2_ is limited by heterogeneous consolidation. These findings highlight that SPS should be regarded as a transformation-assisted consolidation process, where mechanical performance is governed by the coupled evolution of structure, phase stability, and residual defects rather than densification alone.

As shown in Table 4, the hardness achieved in Al_82_Fe_14_Nb_2_Ni_2_ (445.4 HV) compares favorably with related Al-Fe-based amorphous–nanocrystalline alloys consolidated by SPS reported in the literature, confirming the effectiveness of the present processing approach.

From a mechanistic perspective, the observed behavior can be rationalized by the competition between nucleation and growth under SPS conditions, modulated by thermodynamic descriptors (ΔH_mix_ − ΔS_mix_ − δ) and rapid thermal–mechanical coupling. More negative ΔH_mix_ enhances chemical affinity and short-range ordering, increasing the nucleation driving force, while higher configurational entropy (ΔS_mix_) and atomic size mismatch (δ) frustrate long-range atomic rearrangement, suppressing crystal growth. During SPS, the combination of pulsed current, pressure, and rapid heating reduces the time available for diffusion-controlled coarsening, shifting the transformation pathway toward a nucleation-dominated regime.

Within this framework, Al_82_Fe_14_Nb_2_Ni_2_ resides near a kinetic balance where nucleation of fine intermetallic precipitates (e.g., Al_13_Fe_4_, Al_3_Nb) is activated, but subsequent growth is constrained, yielding a fine dispersion within an amorphous/nanocrystalline matrix. In contrast, the quinary alloy, characterized by higher disorder, suppresses both nucleation and growth, leading to enhanced amorphous retention but insufficient precipitation strengthening. The Ti-containing alloy, while readily amorphized during milling, appears to deviate from this balance due to heterogeneous bonding and local diffusion limitations, resulting in incomplete densification and inferior hardness. These results indicate that SPS processing operates within a narrow kinetic window in which the relative rates of nucleation, growth, and densification must be balanced to achieve the highest mechanical performance. This perspective further supports the interpretation of SPS as a transformation-assisted consolidation process, in which microstructural evolution is governed by coupled thermodynamic and kinetic constraints rather than by densification alone.

**Figure 8:**
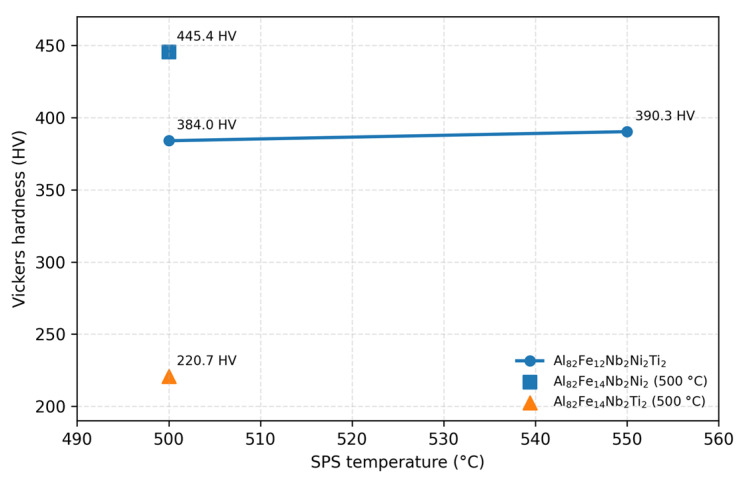
Hardness–temperature response in the investigated SPS window. The quinary Al_82_Fe_12_Nb_2_Ni_2_Ti_2_ alloy was measured at both 500 and 550 °C, whereas the Al_82_Fe_14_Nb_2_Ni_2_ and Al_82_Fe_14_Nb_2_Ti_2_ alloys were measured at 500 °C only.

To make the temperature response explicit without extrapolating beyond the measured conditions, Figure 8 plots the hardness–temperature relationship in the tested SPS window. Because only the quinary alloy was evaluated at both 500 and 550 °C, this plot should be interpreted as a limited two-point temperature response rather than a complete trend map. The slight increase from 384.0 HV at 500 °C to 390.3 HV at 550 °C indicates that moderate additional crystallization provides only limited hardening in the quinary alloy. In contrast, the Ni-containing quaternary alloy sintered at 500 °C still exhibits the highest hardness within the investigated compositions. This comparison reinforces the central conclusion that hardness is controlled by a kinetic optimum involving composition, densification, and devitrification, rather than by temperature or amorphous fraction alone.

**Figure 9:**
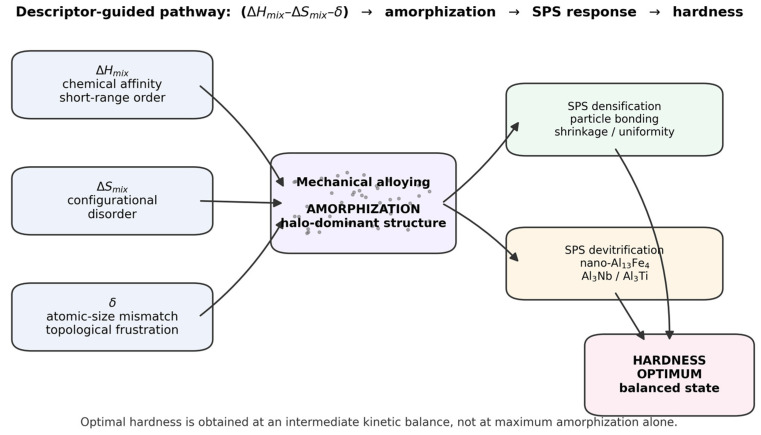
Schematic illustration of the descriptor-guided processing–structure–property relationship.

This descriptor-guided convergence pathway is schematically summarized in Figure 9.

The combined effects of ΔH_mix_, ΔS_mix_, and δ govern amorphization during mechanical alloying, which subsequently controls the densification-devitrification balance during SPS. The highest hardness is achieved at an intermediate state where amorphous retention, fine intermetallic precipitation, and densification are kinetically balanced.

## 4. Conclusions

The present study establishes a processing–structure–property framework for Al-rich Al–Fe–Nb-based metastable alloys modified with Ni and Ti. Mechanical alloying progressively destroys long-range crystalline order in all compositions, while the quinary Al_82_Fe_12_Nb_2_Ni_2_Ti_2_ alloy shows the highest amorphous-forming ability owing to its higher ΔS_mix_ (5.420 J/mol·K) and more negative ΔH_mix_ (−9.36 kJ/mol). At the quaternary level, Ti accelerates amorphization more effectively than Ni, indicating that topological frustration contributes strongly to milling-induced disordering. SPS displacement analysis indicates that the predominant displacement occurs during the heating stage and then approaches a plateau during isothermal holding, reflecting the transition from particle rearrangement and plastic/viscous deformation to diffusion-assisted bonding and crystallization-induced stiffening. SPS at 500 °C produces amorphous–nanocrystalline composites containing a retained amorphous matrix and fine intermetallic precipitates such as Al_13_Fe_4_ and Al_3_Nb, whereas increasing the temperature to 550 °C promotes further devitrification. The highest hardness, 445.4 HV, is obtained for Al_82_Fe_14_Nb_2_Ni_2_ at 500 °C, although this alloy does not exhibit the maximum amorphous-forming ability. Thus, within the investigated SPS window, the highest mechanical performance is associated with an intermediate structural state where amorphous retention, partial nanocrystallization, and densification quality appear to be simultaneously balanced. These findings suggest that SPS operates as a coupled densification–transformation process and offer a preliminary thermodynamic–kinetic basis for designing high-strength Al-rich amorphous–nanocrystalline alloys. The descriptor-guided convergence pathway from ΔH_mix_ − ΔS_mix_ − δ to amorphization, SPS densification/devitrification, and hardness response is summarized in Figure 9.

## Figures and Tables

**Table 1:** Elemental powder masses for preparing 10 g of each alloy by mechanical alloying.

Element	M (g/mol)	Al_82_Fe_14_Nb_2_Ni_2_		Al_82_Fe_14_Nb_2_Ti_2_		Al_82_Fe_12_Nb_2_Ni_2_Ti_2_	
		Mass (g)	wt%	Mass (g)	wt%	Mass (g)	wt%
Al	26.98	6.7094	67.09	6.7538	67.54	6.7421	67.42
Fe	55.85	2.3711	23.71	2.3867	23.87	2.0422	20.42
Nb	92.91	0.5635	5.64	0.5672	5.67	0.5663	5.66
Ni	58.69	0.3560	3.56	-	-	0.3577	3.58
Ti	47.87	-	-	0.2923	2.92	0.2917	2.92
Total		10.000	100	10.000	100	10.000	100

**Table 2:** Calculated thermodynamic and topological parameters, including configurational entropy of mixing (ΔS_mix_), enthalpy of mixing (ΔH_mix_), and atomic-size mismatch (δ), for the investigated Al-based alloys [46].

Element	Radius (rᵢ in Å)	Atomic Pair (i-j)	ΔHAB (kJ/mol)	Alloy Composition	ΔH_mix_ (kJ/mol)	ΔS_mix_ (J/mol·K)	δ (%)
Al	1.43	Al-Fe	−11	Al_82_Fe_14_Nb_2_Ni_2_	−7.92	4.942	4.98
Fe	1.24	Al-Nb	−18	Al_82_Fe_14_Nb_2_Ti_2_	−8.57	4.942	4.73
Nb	1.43	Al-Ni	−22	Al_82_Fe_12_Nb_2_Ni_2_Ti_2_	−9.36	5.420	4.73
Ni	1.24	Al-Ti	−30				
Ti	1.47	Fe-Nb	−16				
		Fe-Ni	−2				
		Fe-Ti	−17				
		Nb-Ni	−30				
		Nb-Ti	+2				

**Table 3:** Average Vickers hardness and standard deviation of SPS-consolidated Al_82_Fe_14_Nb_2_Ni_2_, Al_82_Fe_14_Nb_2_Ti_2_, and Al_82_Fe_12_Nb_2_Ni_2_Ti_2_ alloys sintered at 500 °C and 550 °C under 500 MPa for 5 min.

Sample	Position					Mean Hardness	Standard Deviation
	1	2	3	4	5		
Nb_2_Ni_2_-500	414.09	430.1	425.55	467.05	490.13	445.38	31.93
Nb_2_Ti_2_-500	225.39	268.13	194.52	216.49	198.94	220.69	29.36
Nb_2_Ni_2_Ti_2_-500	362.29	431.96	422.22	311.38	392.38	384.05	48.93
Nb_2_Ni_2_Ti_2_-550	338.16	371.19	448.7	364.65	428.75	390.29	46.45

**Table 4:** Comparison of SPS processing conditions, post-consolidation phases, and Vickers hardness of Al-Fe-based amorphous and amorphous–nanocrystalline alloys reported in the literature and in the present study.

Alloy	Method	SPS Conditions	Phases After SPS	Hardness (HV)	Ref.
Al_82_La_10_Fe_4_Ni_4_	MA + SPS	400 °C, 600 MPa, 5 min	Amorphous + Al_11_La_3_	~350	[29]
Al_86_Ni_8_Y_6_	MA + SPS	100–400 MPa	Retained amorphous matrix + intermetallic nano-precipitates;	1.91–3.56 GPa (~195–363 HV)	[55]
Al_86_Ni_8_Y_6_	MA + SPS	SPS at 300–500 °C	Amorphous matrix + α-Al/nanocrystalline phases	~277 HV	[56]
Al_86_Ni_6_Y_6_Co_2_	MA + SPS	SPS at 300–500 °C	Amorphous/nanocrystalline, more devitrified than ternary alloy	~290 HV	[56]
Al_86_Ni_6_Y_4.5_Co_2_La_1.5_	MA + SPS	SPS at 300–500 °C	Amorphous-dominant	~301 HV	[56]
Al_82_Fe_14_Nb_2_Ni_2_	MA + SPS	500 °C, 500 MPa, 5 min	Amor. + Al_13_Fe_4_ + Al_3_Nb	445.4	This work
Al_82_Fe_14_Nb_2_Ti_2_	MA + SPS	500 °C, 500 MPa, 5 min	Amor. + Al_13_Fe_4_ + Al_3_Nb	220.7	This work
Al_82_Fe_12_Nb_2_Ni_2_Ti_2_	MA + SPS	500 °C, 500 MPa, 5 min	Amor. + Al_13_Fe_4_ + Al_3_Nb	384.1	This work

## Data Availability

The original contributions presented in the study are included in the article/Supplementary Materials; further inquiries can be directed to the corresponding author.

## Supplementary Materials

The following supporting information can be downloaded at: https://www.mdpi.com/article/10.3390/ma19122628/s1; Table S1: Step-by-step calculation of thermodynamic and topological parameters (ΔHₘᵢₓ, ΔSₘᵢₓ, δ) for each alloy composition, following Equations (1)–(4) in the main text. All Miedema binary interaction enthalpies (ΔHᴀᴃ) and atomic radii (rᵢ) are taken from Takeuchi & Inoue, Mater. Trans. 46 (2005) 2817–2829 [46]. Code S1: Python script (table1_calc.py) implementing Equations (1)–(4) for the Miedema-model calculations reported in Table 2. Table S2. PDF/ICDD card numbers and primary reflection positions for phases identified in XRD patterns of mechanically alloyed and SPS-consolidated Al_82_Fe_14−x_Nb_2_(Ni,Ti)_x_ alloys.
