# Supplementary material for: Amorphization–Densification Coupling Governs Hardness Enhancement in SPS-Consolidated Al–Fe–Nb–(Ni,Ti) Metastable Alloys
Source: Materials (Basel). 2026 Jun 18;19(12):2628. doi: 10.3390/ma19122628 (PMC13303422; doi:10.3390/ma19122628)
Supplement: Supplementary file 1 [file materials-19-02628-s001.zip › Table_S1_S2.pdf]

Supplementary Materials

Amorphization–Densification Coupling Governs Hardness Enhancement in SPS-Consolidated Al–Fe–Nb–(Ni,Ti) Metastable Alloys

Table S1.

Step-by-step calculation of thermodynamic and topological parameters (ΔH_{mix}, ΔS_{mix}, δ) for each alloy composition, following Equations (1)–(4) in the main text. All Miedema binary interaction enthalpies (ΔH_{AB}) and atomic radii (r_i) are taken from Takeuchi & Inoue, Mater. Trans. 46 (2005) 2817–2829.

1. $Al_{82}Fe_{14}Nb_2Ni_2$

Part A – Enthalpy of mixing (ΔH_{mix})

$\Delta H_{mix} = \Sigma\,(i\neq j)\; 4\cdot\Delta H_{AB}\cdot C_i\cdot C_j$

Atomic pair (i–j)	ΔH_{AB} (kJ mol⁻¹)	C_i	C_j	$4\cdot\Delta H_{AB}\cdot C_i\cdot C_j$ (kJ mol⁻¹)
Al–Fe	-11	0.820	0.140	-5.0512
Al–Nb	-18	0.820	0.020	-1.1808
Al–Ni	-22	0.820	0.020	-1.4432
Fe–Nb	-16	0.140	0.020	-0.1792
Fe–Ni	-2	0.140	0.020	-0.0224
Nb–Ni	-30	0.020	0.020	-0.0480
**ΔH_{mix} = -7.9248 kJ mol⁻¹**				

Part B—Configurational mixing entropy (ΔS_{mix})

$\Delta S_{mix} = -R\cdot\Sigma\, C_i\cdot \ln(C_i)$

Element	C_i	$C_i\cdot\ln(C_i)$	$\Delta S^i = -R\cdot C_i\cdot\ln(C_i)$ (J mol⁻¹ K⁻¹)
Al	0.820	-0.162730	1.3529
Fe	0.140	-0.275256	2.2885
Nb	0.020	-0.078240	0.6505
Ni	0.020	-0.078240	0.6505
**ΔS_{mix} = 4.9424 J mol⁻¹ K⁻¹**			

Part C—Atomic-size mismatch (δ)

$\delta = \sqrt{[\Sigma\, C_i\cdot(1-r_i/\bar{r})^2]} \times 100\%$

Element	C_i	r_i (Å)	$r_i / \bar{r}$	$(1-r_i/\bar{r})^2$	$C_i\cdot(1-r_i/\bar{r})^2$
Al	0.820	1.430	1.021720	0.00047178	0.00038686
Fe	0.140	1.240	0.885967	0.01300343	0.00182048
Nb	0.020	1.430	1.021720	0.00047178	0.00000944
Ni	0.020	1.240	0.885967	0.01300343	0.00026007
**$\bar{r}$ = 1.3996 Å $\Sigma\, C_i\cdot(1-r_i/\bar{r})^2$ = 0.00247684 δ = 4.9768 %**					

2. $Al_{82}Fe_{14}Nb_2Ti_2$

Part A—Enthalpy of mixing (ΔH_{mix})

$\Delta H_{mix} = \Sigma\,(i\neq j)\; 4\cdot\Delta H_{AB}\cdot C_i\cdot C_j$

Atomic pair (i–j)	ΔH_{AB} (kJ mol⁻¹)	C_i	C_j	$4\cdot\Delta H_{AB}\cdot C_i\cdot C_j$ (kJ mol⁻¹)
Al–Fe	-11	0.820	0.140	-5.0512
Al–Nb	-18	0.820	0.020	-1.1808

Atomic pair (i–j)	ΔH_{AB} (kJ mol⁻¹)	C_i	C_j	$4\cdot\Delta H_{AB}\cdot C_i\cdot C_j$ (kJ mol⁻¹)
Al–Ti	-30	0.820	0.020	-1.9680
Fe–Nb	-16	0.140	0.020	-0.1792
Fe–Ti	-17	0.140	0.020	-0.1904
Nb–Ti	2	0.020	0.020	0.0032
**ΔH_{mix} = -8.5664 kJ mol⁻¹**				

Part B—Configurational mixing entropy (ΔS_{mix})

$\Delta S_{mix} = -R\cdot\Sigma\, C_i\cdot\ln(C_i)$

Element	C_i	$C_i\cdot\ln(C_i)$	$\Delta S^i = -R\cdot C_i\cdot\ln(C_i)$ (J mol⁻¹ K⁻¹)
Al	0.820	-0.162730	1.3529
Fe	0.140	-0.275256	2.2885
Nb	0.020	-0.078240	0.6505
Ti	0.020	-0.078240	0.6505
**ΔS_{mix} = 4.9424 J mol⁻¹ K⁻¹**			

Part C—Atomic-size mismatch (δ)

$\delta = \sqrt{[\Sigma\, C_i\cdot(1-r_i/\bar{r})^2]} \times 100\%$

Element	C_i	r_i (Å)	$r_i / \bar{r}$	$(1-r_i/\bar{r})^2$	$C_i\cdot(1-r_i/\bar{r})^2$
Al	0.820	1.430	1.018373	0.00033758	0.00027682
Fe	0.140	1.240	0.883065	0.01367377	0.00191433
Nb	0.020	1.430	1.018373	0.00033758	0.00000675
Ti	0.020	1.470	1.046859	0.00219581	0.00004392
**$\bar{r}$ = 1.4042 Å $\Sigma\, C_i\cdot(1-r_i/\bar{r})^2$ = 0.00224181 δ = 4.7348 %**					

3. $Al_{82}Fe_{12}Nb_2Ni_2Ti_2$

Part A—Enthalpy of mixing (ΔH_{mix})

$\Delta H_{mix} = \Sigma\,(i\neq j)\, 4\cdot\Delta H_{AB}\cdot C_i\cdot C_j$

Atomic pair (i–j)	ΔH_{AB} (kJ mol⁻¹)	C_i	C_j	$4\cdot\Delta H_{AB}\cdot C_i\cdot C_j$ (kJ mol⁻¹)
Al–Fe	-11	0.820	0.120	-4.3296
Al–Nb	-18	0.820	0.020	-1.1808
Al–Ni	-22	0.820	0.020	-1.4432
Al–Ti	-30	0.820	0.020	-1.9680
Fe–Nb	-16	0.120	0.020	-0.1536
Fe–Ni	-2	0.120	0.020	-0.0192
Fe–Ti	-17	0.120	0.020	-0.1632
Nb–Ni	-30	0.020	0.020	-0.0480
Nb–Ti	2	0.020	0.020	0.0032
Ni–Ti	-35	0.020	0.020	-0.0560
**ΔH_{mix} = -9.3584 kJ mol⁻¹**				

Part B—Configurational mixing entropy (ΔS_{mix})

$\Delta S_{mix} = -R\cdot\Sigma\, C_i\cdot\ln(C_i)$

Element	C_i	$C_i\cdot\ln(C_i)$	$\Delta S^i = -R\cdot C_i\cdot\ln(C_i)$ (J mol^{-1} K^{-1})
Al	0.820	-0.162730	1.3529
Fe	0.120	-0.254432	2.1153
Nb	0.020	-0.078240	0.6505
Ni	0.020	-0.078240	0.6505
Ti	0.020	-0.078240	0.6505
**ΔS_{mix} = 5.4197 J mol^{-1} K^{-1}**			

Part C—Atomic-size mismatch (δ)

$\delta = \sqrt{[\Sigma\, C_i\cdot(1-r_i/\bar{r})^2]} \times 100\%$

Element	C_i	r_i (Å)	$r_i / \bar{r}$	$(1-r_i/\bar{r})^2$	$C_i\cdot(1-r_i/\bar{r})^2$
Al	0.820	1.430	1.018373	0.00033758	0.00027682
Fe	0.120	1.240	0.883065	0.01367377	0.00164085
Nb	0.020	1.430	1.018373	0.00033758	0.00000675
Ni	0.020	1.240	0.883065	0.01367377	0.00027348
Ti	0.020	1.470	1.046859	0.00219581	0.00004392
**$\bar{r}$ = 1.4042 Å $\Sigma\, C_i\cdot(1-r_i/\bar{r})^2$ = 0.00224181 δ = 4.7348 %**					

Summary—Parameters for Table 2 (main text)

Alloy composition	ΔH_{mix} (kJ mol^{-1})	ΔS_{mix} (J mol^{-1} K^{-1})	δ (%)
$Al_{82}Fe_{14}Nb_2Ni_2$	-7.92	4.942	4.98
$Al_{82}Fe_{14}Nb_2Ti_2$	-8.57	4.942	4.73
$Al_{82}Fe_{12}Nb_2Ni_2Ti_2$	-9.36	5.420	4.73

Reference: Takeuchi, A.; Inoue, A. Classification of Bulk Metallic Glasses by Atomic Size Difference, Heat of Mixing and Period of Constituent Elements. Mater. Trans. 2005, 46, 2817–2829.

Table S2. PDF/ICDD card numbers and primary reflection positions for phases identified in XRD patterns of mechanically alloyed and SPS-consolidated $Al_{82}Fe_{14-x}Nb_2(Ni,Ti)_x$ alloys.

Phase	Crystal structure	Space group	PDF2 card No.*	Primary 2θ (°)	hkl
Al	*fcc*	$Fm\bar{3}m$	04-0787	38.47, 44.74, 65.13	111, 200, 220
Fe	*bcc*	$Im\bar{3}m$	06-0696	44.67, 65.02, 82.33	110, 200, 211
Nb	*bcc*	$Im\bar{3}m$	35-0789	38.47, 55.48, 69.59	110, 200, 211
Ni	*fcc*	$Fm\bar{3}m$	04-0850	44.51, 51.85, 76.37	111, 200, 220
Ti (α)	*hcp*	$P6_3/mmc$	44-1294	35.09, 38.42, 40.17	100, 002, 101
$Al_{13}Fe_4$	*monoclinic*	C2/m	29-0042	44.14, 62.73, 64.18	–
Al_3Nb / $NbAl_3$	*tetragonal*	I4/mmm	13-0146	20.59, 25.35, 39.17, 41.95, 47.31, 65.03	002, 101, 112, 004, 200, –
Al_3Ti	*tetragonal*	I4/mmm	37-1449	39.12, 47.13, 82.20	112, 200, 303

** PDF card numbers as identified by MDI Jade 6.5 software using the PDF-2 database. The listed 2θ values correspond to Cu Kα radiation (λ = 1.5406 Å). Minor peak shifts may occur because of lattice strain, solid-solution formation, and nanoscale crystallite size after mechanical alloying and SPS. For complex intermetallic phases such as $Al_{13}Fe_4$, peak assignment was used mainly for phase identification because several reflections overlap with Al, Fe, Nb, and Ni peaks.*
